# Pectosomes and Chitosomes as Delivery Systems for Metronidazole: The One-Pot Preparation Method

**DOI:** 10.3390/pharmaceutics5030445

**Published:** 2013-09-06

**Authors:** Toril Andersen, Željka Vanić, Gøril Eide Flaten, Sofia Mattsson, Ingunn Tho, Nataša Škalko-Basnet

**Affiliations:** 1 Drug Transport and Delivery Research Group, Department of Pharmacy, Faculty of Health Sciences, University of Tromsø, Tromsø 9037, Norway; E-Mails: toril.andersen@uit.no (T.A.); goril.flaten@uit.no (G.E.F.); ingunn.tho@uit.no (I.T.); 2 Department of Pharmaceutics, Faculty of Pharmacy and Biochemistry, University of Zagreb, Zagreb 10000, Croatia; E-Mail: zeljka.vanic@pharma.hr; 3 Department of Clinical Pharmacology, Umeå University, Umeå SE-90185, Sweden; E-Mail: sofia.mattsson@pharm.umu.se

**Keywords:** liposomes, pectosomes, chitosomes, metronidazole, mucoadhesion, polymer-coating, vaginal therapy

## Abstract

Mucoadhesive liposomes offer a potential for improved residence time of liposomal systems targeting contact with mucosal tissues, such as in buccal, oral, colon, and vaginal drug delivery. Most of the currently available methods rely on the coating of preformed liposomes by various mucoadhesive polymers. The aim of this study was to develop novel mucoadhesive system by the one-pot preparation method. The pectin- and chitosan-containing liposomes, namely pectosomes and chitosomes, were prepared by the modified solvent injection method. In order to optimize this novel delivery system, we used pectins and chitosans of both high and low degree of esterification/deacetylation (DE/DD), respectively. Sonication was applied to reduce the original vesicle size. All vesicles were characterized for their size, zeta potential, metronidazole entrapment, and stability. Both pectosomes and chitosomes were found to entrap more metronidazole than conventional plain liposomes. Preliminary data indicate that the polymer is present on the liposomal surface, embedded within inner liposomal bilayers, and entrapped inside the aqueous compartment. The next step in the evaluation of this system is the testing of its mucoadhesiveness.

## 1. Introduction

Bacterial vaginosis, the most common vaginal infection in women of childbearing age, and is often treated by local administration of metronidazole rather than systemically [1]. However, most of the conventional dosage forms are limited in the retention time at the vaginal site, often failing to achieve intended therapy outcome. Several classes of nanopharmaceuticals have been proposed as a mean to overcome the limitations of conventional dosage forms [2]. Our particular interest was mucoadhesion as a means of prolonging vaginal residence time. The mucoadhesive nanopharmaceuticals could ensure prolonged and intimate contact with the mucus, thus enhancing the delivery of drugs to the underlying tissue. Moreover, nanopharmaceuticals could also provide sustained and controlled drug release [3]. The surface properties of mucoadhesive nanopharmaceuticals play an important role in their retention and delivery capacities once they come in contact with the vaginal mucosa and are, therefore, crucial for successful drug therapy [2].

To prepare mucoadhesive nanopharmaceuticals, we have selected two natural polymers of confirmed mucoadhesiveness, namely chitosan and pectin [4]. Both polymers are available in the different molecular weights and degrees of deacetylation/esterification (DD/DE), which are known to affect their physicochemical properties and may have an impact on their mucoadhesiveness [5,6].

Pectin is a polysaccharide obtained from apple pomace or citrus peel. Its structure and properties vary with the source and the conditions applied during isolation. The acid groups of the galacturonic units can be methoxylated and/or amidated to varying degrees. The degree of esterification (DE) is expressed as a percentage of carboxylic groups that carry an ester and will determine both its physical and chemical properties. Pectin is biodegradable, biocompatible, and non-toxic and is therefore a promising polymer for mucoadhesive drug delivery systems [7,8].

Chitosan is obtained by *n*-deacetylation of chitin; a polysaccharide found in the shells of shrimps, consisting of glucosamine and *N*-acetylglucosamine units. Chitosan interacts well with mucin, one of the principal components of mucus. Its main advantages are conformed biodegradability and biocompatibility, as well as the ability to form gels [5].

Mucoadhesive nano- and micro-pharmaceuticals have been prepared as various delivery systems, such as for example microbeads [9], nanoparticles [10], nanoemulsions [11], and polymer-coated liposomes [12,13]. Liposomes have been studied for over 40 years as drug delivery systems for various routes of drug administration, including the vaginal route [2]. The concept of the coating of the liposomal surface with a mucoadhesive polymer has been proposed, relatively early, as a means to increase the retention time on the mucosal surface with a specific target being the intestinal surface [14]. However, most of the currently available methods for the preparation of mucoadhesive liposomes rely on the coating of preformed liposomes by various mucoadhesive polymers [12,13,15].

The aim of the current study was to develop a novel mucoadhesive system that would allow for straightforward and simple preparation procedure, such as, for example, a one-pot preparation method. Pectin- and chitosan-containing liposomes were prepared by a modified solvent injection method where the dissolved lipid film was injected into diluted solutions of pectin or chitosan. The original vesicle size was reduced by sonication. To optimize the delivery system, pectins and chitosans, of both high and low degree of esterification/deacetylation (DE/DD), were evaluated. Although our current focus is on the topical vaginal therapy, metronidazole [2-(2-methyl-5-nitro-1*H*-imidazol-1-yl)ethanol], as model drug, can also be used in oral or buccal therapy. Metronidazole exhibits its antimicrobial activity through reduction of its nitro group and the formation of toxic derivatives, inducing the death of susceptible microorganisms through interacting with DNA. In addition, metronidazole is characterized by a low solubility, both in water and in organic solvents, and is therefore challenging to formulate, particularly in topical formulations [16]. All formulations were characterized with respect to their size, zeta potential, metronidazole entrapment, and stability.

## 2. Experimental Section

### 2.1. Materials

Soy phosphatidylcholine (Lipoid S100, Lipoid GmbH, Ludwigshafen, Germany) was a generous gift by Lipoid GmbH. Chitosan of varying degrees of deacetylation, Fiske-SubbaRow reducer, metronidazole, methanol, *n*-propanol, and phosphorus standard solution were purchased from Sigma Aldrich Inc. (St. Luis, MO, USA). Pectins of varying degree of esterification were the product of Herbstreith & Fox KG (Neuenbürg, Germany). Ammonium molybdate and peroxide were purchased from Merck KGaA, (Darmstadt, Germany), while sulphuric acid was the provided by May and Baker LTD (Dagenham, England). All other chemicals used in the experiments were of analytical grade.

### 2.2. Viscosity of Polymer Solutions

The viscosity of the aqueous solutions of polymers was measured using a rotational viscometer (Haake Viscotester 7 plus, Thermo Electron GmbH, Karlsruhe, Germany) with a TL 5 spindle. The polymer concentrations of the different polymers were adjusted to the viscosity of the aqueous solution in the range of 0.65 ± 0.20 mPas. Samples were tested in triplicate. 

### 2.3. Preparation of Liposomes

Liposomes were prepared by the modified method of solvent injection originally described by Gentine *et al*. [17]. In brief, Lipoid S100 (SPC, 200 mg), and 20 mg of metronidazole were dissolved in methanol. The solvent was evaporated using a rotoevaporator system (Büchi rotavapor R-124 with vacuum controller B-721, Büchi Vac V-500, Büchi Labortechnik, Flawil, Switzerland) under a vacuum at 45 °C. The resulting lipid film was redispersed in 100 μL of *n*-propanol with a micro-syringe pipette (Hamilton Company, Bonaduz, Switzerland). The dispersion was injected via a needle into 2 mL of aqueous polymer solution of pectin or chitosan, and stirred for 2 h at room temperature. Two grades of pectins with different degrees of esterification (35% and 50% DE, respectively) were used, as well as the two grades of chitosan with different degrees of deacetylation (77% and 95% DD, respectively). The respective polymer concentrations were adjusted to be of a similar viscosity as described above (2.2), resulting in the final concentration of 0.50% (*w*/*w*) aqueous solution of each of the two pectins, and 0.05% (*w*/*w*) of chitosan DD 77% and 0.17% of chitosan DD 95% in 0.1% (*v*/*v*) acetic acid, respectively. Plain liposomes of the same lipid composition, and prepared under the same conditions, served as control. We also prepared polymer-coated liposomes (preformed liposomes containing the drug and coated as described in [13] for a comparison, however, due to low metronidazole loading, these data were not included).

### 2.4. Vesicle Size Reduction

The size of liposomes was reduced by the sonication using a Sonics High Ultrasonic Processor (Sigma-Aldrich Chemie GmgH, Steinheim, Germany). The samples were sonicated for either 1 or 2 min (1 + 1 min) using an ice bath to prevent heating of the samples. The apparatus was allowed a period of cooling down in between sonication runs of about 2–3 min.

### 2.5. Entrapment Efficiency

To remove unentrapped drug from the polymer-containing liposomal dispersions, chitosomes and pectosomes were dialyzed against distilled water for 4 h at room temperature (Mw cut off: 12–14,000 Daltons; Medicell International Ltd., London, UK). The volume was adjusted to assure the sinks conditions.

The amount of drug entrapped in the liposomal formulations was determined by UV spectrophotometry (Agilent Technologies, Santa Clara, CA, USA). Liposomal samples were dissolved in methanol and metronidazole content measured at 311 nm. The standard curves of metronidazole in methanol were prepared using the concentrations ranging from 2 to 20 μg/mL (*R*^2 ^= 0.9999).

### 2.6. Phosphorus Assay

The content of phosphatidylcholine (PC) was measured using the modified Bartlett method [18]. In brief, the samples were diluted to appropriate concentration in distilled water and an aliquot (1 mL) mixed with 0.5 mL of 10 N H_2_SO_4_ and heated at 160 °C for a minimum of 3 h. After the cooling, 2 drops of 30% (*v*/*v*) H_2_O_2_ was added and the mixture heated to 160 °C for 1.5 h. The ammonium molybdate (4.6 mL; 0.22% *v*/*v*) and 0.2 mL of Fiske-SubbaRow reagent were added after the cooling, mixed, and the mixture was heated for 7 min at 100 °C. All samples were analyzed by UV spectrophotometry at 830 nm. The phosphorus standard solution was used to prepare a standard curve.

### 2.7. Particle Size Analysis

The particle size distributions of the non-sonicated polymer-containing liposomes were determined by photon correlation spectroscopy (PCS) on Zetasizer 3000HS (Malvern Instruments, Malvern, UK). The measurements were performed at a scattering angle of 90° and a temperature of 25 °C. The dispersions were diluted with 1 mM NaCl, which was previously filtered through 200 nm Minisart filters, to achieve a count rate between 100 and 300 kcps [16].

The morphology and particle size distributions (based on the number of particles) of the non-sonicated polymer-containing liposomes were also estimated with the aid of an Olympus BH-2 microscope equipped with a computer-controlled image analysis system (Optomax V, Cambridge, UK). In all measurements 1000 particles were examined [19].

After size reduction of the polymer-containing liposomes, the particle size distributions of all liposomal dispersions were measured by PCS using a Submicron particle-sizer (model 370, Nicomp, Santa Barbara, CA, USA). The samples were diluted with filtered (0.2 μm Millipore filters) distilled water until the appropriate count rate (approximately 250–350 kHz) and measured in triplicate. The polydispersity index (PI) and the average diameter were used to characterize the samples [20].

### 2.8. Zeta Potential

The zeta potential of the non-sonicated and sonicated polymer-containing liposomes was measured with a Nano ZS (Malvern Instruments Ltd, Worcestershire, UK). All samples were diluted in filtered water until an appropriate concentration was achieved and measured in a measuring cell. All results are presented as the average of at least three independent measurements for each liposome formulation.

### 2.9. pH Measurements 

pH was determined both in the polymer solutions and the dispersion of polymer-containing liposomes in order to elucidate whether, and to what extent, an interaction between polymer and liposomes took place. A change in pH of the polymer solution after the injection of dissolved lipids may be interpreted as a “loss” of dissolved polymer, *i.e.*, polymer could be entrapped or closely associated with the liposomes. pH was measured at 22 °C using a calibrated pH meter (Metrohm AG, Herisau, Switzerland). 

### 2.10. Stability Testing

The stability of the newly developed liposomes was determined after one month of storage in a refrigerator (4 °C). All liposomes were tested for the entrapment (retention of the originally entrapped metronidazole), size distributions, and zeta potential.

## 3. Results and Discussion

### 3.1. Viscosity of the Polymer Solutions

To minimize the effect of different polymer viscosities on the formation of liposomes, the viscosity of each polymer solution was determined and the concentrations were adjusted so that the viscosities of the polymer solutions used in the preparation of liposomes were in a similar range (0.65 ± 0.20 mPas). An overview of the employed viscosities and the corresponding concentration of the polymers can be seen in Table 1.

**Table 1:** Characteristics of the polymer solutions (*n* = 3).

Polymer type	Conc. (%, *w*/*w*)	Viscosity (mPas)
Pectin (35% DE)	0.50	0.85
Pectin (50% DE)	0.50	0.67
Chitosan (77% DD)	0.17	0.75
Chitosan (95% DD)	0.05	0.49

### 3.2. Entrapment Efficiency

**Figure 1:**
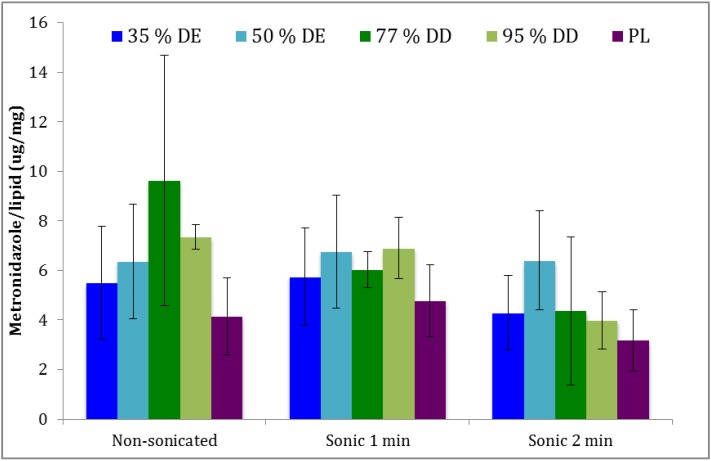
Entrapment efficiency of metronidazole in the different liposomal formulations. Pectosomes (35% and 50% DE) are labeled in blue, chitosomes (77% and 95%DD) green, and the plain liposomes (PL) in purple (*n* = 3).

To achieve a successful local treatment of bacterial vaginosis, it is very important to assure that a sufficient amount of the drug remains at the vaginal site over a required period of time [1]. Any mucoadhesive nanopharmaceutical destined to vaginal administration needs to carry a sufficient quantity of drug and release it in a predictable manner [2]. When designing and preparing the new type of mucoadhesive vesicles, we wanted to simplify the preparation procedure by minimizing the steps required for the preparation of mucoadhesive vesicles, particularly the dilution step involved in the conventional coating. Therefore, as comparison, we originally prepared polymer-coated liposomes by the conventional approach, namely coating of preformed liposomes [13]. However, the encapsulation efficiencies for metronidazole in liposomes were all very low (below 5%; data not shown), and the subsequent coating of liposomes resulted in further dilution and reduction of the concentration of therapeutically available metronidazole. To prepare mucoadhesive liposomes, containing either pectin or chitosan and carrying sufficient drug load, we developed the new approach and vesicle preparation method, which is a modification of the recently reported solvent injection method [17]. The novelty of our method lies in the procedure based on the addition of the polymer solution prior to the formation of liposomes, resulting in liposome coating *in situ*. To the best of our knowledge, this is a novel approach in preparing polymer-containingvesicles. In mucoadhesivevesicles, the polymer is expected to be both vesicle-entrapped and surface-available, as some of the polymer will be encapsulated in the aqueous compartments of vesicle. The entrapment of metronidazole (Figure 1) in the different formulations was presented as the amount of drug per lipid, normalized after determination of the lipid amount in each formulation as determined by the phosphor assay. It is known that the vesicle size plays an important role in the ability of nanodelivery system to reach the underlying tissue within vaginal cavity, having a direct impact on the success of the therapy [3]. Therefore, we prepared and compared the potential of the non-sonicated and two types of sonicated mucoadhesive formulations to deliver sufficient amount of metronidazole (Figure 1).

The entrapment in both pectosomes and chitosomes was found to be higher than in the plain liposomes, although not on a significant level, due to a relatively larger SD. The sonication for 1 min did not lead to loss of originally entrapped metronidazole, however longer sonication (2 min) resulted in smaller liposomes (Table 2), carrying less of the originally entrapped drug, as expected. It appeared that chitosomes were superior to pectosomes with respect to the entrapment of metronidazole (Figure 1). However, again, the differences were not significant. Interestingly, pectosomes prepared from pectin with 50% DE appeared to be the most stable regarding the loss of the originally entrapped metronidazole, a fact which needs to be further evaluated. Chitosomes, on the other hand, seemed to lose more of the originally associated drug during sonication.

### 3.3. Characteristics of Vesicles

Size determination for non-sonicated vesicles revealed that vesicles were larger than one micron, regardless of their composition or the presence of polymer (data not shown). The polydispersity indexes for those formulations were over 0.70, therefore, we assumed that mulitilamellar vesicles were formed and their sizes can be taken only as an estimate rather than the absolute values. Although the original isopropanol injection method [17] was developed to manufacture unilamellar vesicles, the presence of polymer onto/into liposomes resulted in larger size. Due to the high polydispersity of vesicle suspension, we applied image analysis to gain a deeper insight on the possible aggregation of the vesicles and their overall structures. However, the image analysis only confirmed that the vesicles were larger than one micron. The vesicle shape and structure was found to be similar for all formulations, although one has to take into the account that image analysis is flattening the 3D structure and that all vesicles appear spherical. The size and size distributions of sonicated vesicles, both freshly prepared and those stored for one month, are presented in Table 2. The size distributions are expressed as NICOMP distributions, *i.e.*, a bimodal distribution where particles of similar size are grouped in populations and presented as the percentage of particles with the specific mean diameter [20]. The sonication of all types of vesicles resulted in vesicles of smaller size. A very interesting phenomenon has been observed with pectosomes prepared from pectin with 50% DE; in this case, longer sonication did not result in smaller vesicles, as observed for the other types of liposomes. Those vesicles were also defined by the minimum loss of the originally incorporated metronidazole (Figure 1), which indicates that this pectin grade stabilizes the vesicles and protects the original vesicle structure during the stress caused by sonication. It would be very interesting to explore these vesicles further and we are currently working on their detailed characterization. Chitosomes prepared from 77% DD chitosan were the smallest of the polymer-coated vesicles (Table 2). Those vesicles also lost more of the originally incorporated drug during the sonication process than the other vesicles (Figure 1).

**Table 2:** Vesicle sizes (*n*=3).

Type of liposomes	Sonication time	Peak 1		Peak 2		PI
		Size (nm)	%	Size (nm)	%	
*Freshly prepared*						
Pectosomes (35% DE)	1 min	148	13	626	86	0.373
	2 min	91	14	324	84	0.287
Pectosomes (50% DE)	1 min	222	11	847	82	0.446
	2 min	166	12	718	86	0.397
Chitosomes (77% DD)	1 min	62	30	239	72	0.315
	2 min	58	21	193	76	0.384
Chitosomes (95% DD)	1 min	194	14	733	86	0.442
	2 min	67	10	290	91	0.421
Plain	1 min	91	18	450	83	0.329
	2 min	82	12	415	89	0.446
*Stored for 1 month*						
Pectosomes (35% DE)	1 min	115	14	497	85	0.324
	2 min	69	14	265	85	0.275
Pectosomes (50% DE)	1 min	113	10	508	90	0.390
	2 min	126	16	473	83	0.347
Chitosomes (77% DD)	1 min	68	23	310	75	0.360
	2 min	122	29	410	69	0.320
Chitosomes (95% DD)	1 min	115	6	625	93	0.525
	2 min	108	3	774	98	0.217
Plain	1 min	69	15	316	85	0.367
	2 min	47	7	222	93	0.454

**Figure 2:**
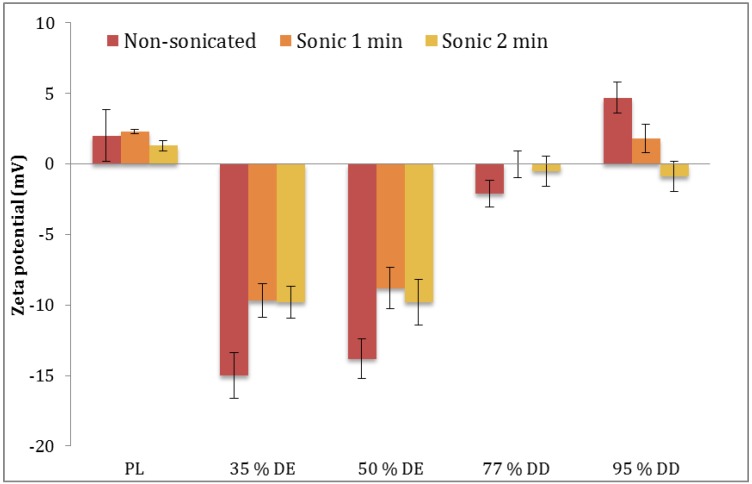
Zeta potential of different liposomes in relation to applied sonication time. PL, plain liposomes; pectosomes (35% and 50% DE); chitosomes (77% and 95% DD) (*n* = 3).

Regarding the stability of vesicles with respect to their size distributions, stored vesicles appeared to be smaller than the freshly prepared vesicles. Although contradictory at first glance, we have observed similar behavior with curcumin-containing vesicles [21]; this could be explained by the stabilization of vesicles during the storage at cold temperature and the fact that the measurements of freshly prepared vesicles are actually overestimates and are including agglomerates rather than separated vesicles. The stored chitosomes sonicated for 2 min appear to aggregate and exhibit larger mean diameters than freshly prepared liposomes of the same type (Table 2), which may be contributed to possible loss of the protecting polymer layer on the vesicle surface during sonication and the consequent changes in zeta potential (Figure 2).

The zeta potential of the polymer-containing liposomes and plain liposomes has been determined to provide information on the changes in vesicles surface charge due to the presence of polymer onto/into vesicles (Figure 2). It is evident that the zeta potential of polymer-containing liposomes is changed as a result of the sonication, which could be explained as a consequence of a removal of the charged polymer, from the vesicle outer bilayer, as confirmed for pectosomes. However, in the case of chitosomes, we need to further evaluate the effect of sonication on the change in vesicle surface charge. The change in zeta potential is also an indirect indication that polymer was present on the vesicle surface and that less of a polymer is exposed on the outer layers in the sonicated vesicles as compared to the non-sonicated vesicles. The lipid used, SPC, is a neutral lipid, therefore the zeta potential of polymer-containing vesicles can be attributed to the charged polymers.

Additional evidence of the presence of polymer on/inside liposomes can be seen through the comparison of the pH values of polymer solutions *versus* the pH of the polymer-containing liposomal dispersions. Whereas the pectin solutions had pH values of 2.93 (35% DE) and 3.05 (50% DE), respectively, the pH of pectosome suspensions was 3.31 (35% DE) and 3.42 (50% DE), respectively. Similarly, the pH of chitosan solutions was 3.43 (77% DD) and 3.78 (95% DD), respectively, and chitosomal suspensions exhibited the pH of 3.91 (77% DD) and 4.26 (95% DD), respectively. All polymer-containing vesicle suspensions had a lower pH than the pH of the plain liposomal suspension, which was close to pH of 5.5. The increase in pH observed in the pectin solutions upon formation of pectosomes might also be taken as a proof that the polymer is also embedded in the vesicles and not only surface-available.

### 3.4. Storage Stability

**Figure 3:**
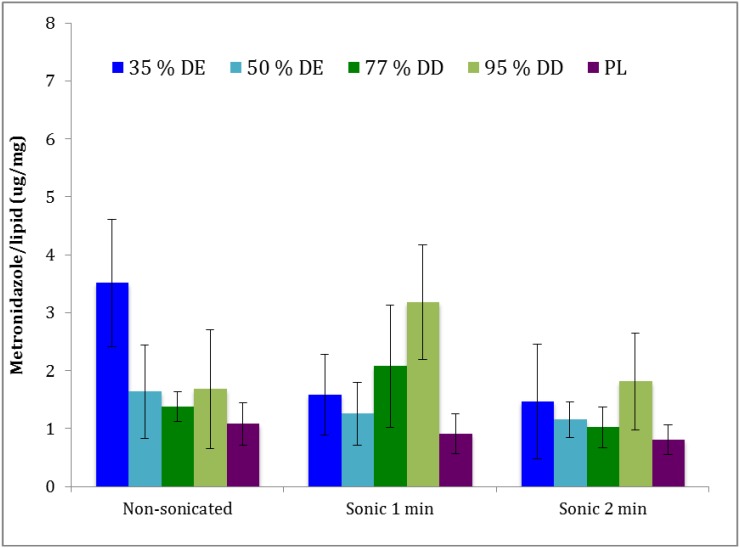
Liposomally-retained metronidazole upon storage for one month at 4 °C. Pectosomes (35% and 50% DE) are labeled in blue, chitosomes (77% and 95% DD) green, and the plain liposomes (PL) in purple (*n* = 3).

Figure 3 represents the drug retention values for the liposomally-associated metronidazole after storage for one month at 4 °C. The corresponding vesicle size distributions are presented in Table 2. Although polymer-containing liposomes retained more of the originally incorporated metronidazole, the differences were not on a significant level.

The potential problem of instability of newly developed vesicles upon storage in the cold conditions requires further testing at various temperatures and should be evaluated for the drugs of different lipophilicities.

## 4. Conclusions

The preparation process reported herein is simple and straightforward and has a potential to be used in the manufacturing of liposomes. The method can be applied for various types of polymers and possibly also polymer combinations.

Moreover, the method reduces the time required to manufacture mucoadhesive formulation. The newly developed vesicles were able to incorporate the sufficient amount of model antimicrobial agent. We are extensively characterizing the structure of the delivery systems contained in these formulations and trying to use the same approach to develop mucoadhesive nanopharmaceuticals for different antimicrobials interesting for vaginal administration. The real potential of the new type of vesicles remains to be confirmed in suitable *in vitro* and *in vivo* model.
